# Effect of catgut implantation at acupoints for the treatment of allergic rhinitis: a randomized, sham-controlled trial

**DOI:** 10.1186/s12906-016-1400-x

**Published:** 2016-11-10

**Authors:** Xinrong Li, Yang Liu, Qinxiu Zhang, Nan Xiang, Miao He, Juan Zhong, Qing Chen, Xiaopei Wang

**Affiliations:** 1 Department of Otorhinolaryngology, Head and Neck Surgery of the Teaching Hospital of Chengdu University of Traditional Chinese Medicine, Chengdu, Sichuan Province 610072 People’s Republic of China; 2 Chengdu University of Traditional Chinese Medicine, Chengdu, Sichuan Province 610072 People’s Republic of China

**Keywords:** Allergic rhinitis, Catgut implantation at acupoints, Randomized controlled trial

## Abstract

**Background:**

The effect and safety of catgut implantation at acupoints o treat allergic rhinitis (ICD-10 code J30.4) remain controversial. Here, we used a sham catgut implantation group to determine whether catgut implantation at acupoints is an effective and safe treatment for allergic rhinitis.

**Methods:**

A randomized double-blind clinical trial, with parallel groups was conducted. Skin prick and puncture test (SPT) was performed to confirm the diagnosis before enrollment. The participants received two sessions of treatments of active or sham catgut implantation at acupoints (once every two weeks) with a follow-up phase of 8 weeks. The visual analogue scale (VAS) and Rhinoconjunctivitis Quality of Life Questionnaire (RQLQ) were used to determine the severity of allergic rhinitis. The use of anti-allergic medication was used as a secondary indicator. The incidence of adverse events was also recorded and analyzed.

**Results:**

An improvement of the VAS and RQLQ scores was observed in both the active and sham-controlled group sat four and eight weeks after the treatment in the self-control analysis. Comparison revealed no significant difference between the treatment and sham-controlled groups until 8 weeks after the 2-week treatment regimen (*t* = −2.424, *P* = 0.017). However, the RQLQ scores significantly differed between the two groups after 4 weeks of treatment completion (*t* = −2.045, *P* = 0.05) and this difference lasted until the end of 8-week follow-up (*t* = −2.246, *P* = 0.033). Throughout the treatment regimen, none of the participants took any relief medication, and no severe adverse events occurred.

**Conclusion:**

Our findings suggest that catgut implantation at acupoints is an effective and safe method for symptomatic treatment of allergic rhinitis.

**Trial registration:**

Chinese Clinical Trial Registry ChiCTR-TRC-12002191 (Date of Registration: 2012-05-09)

## Background

Allergic rhinitis (ICD-10 code J30.4) is a symptomatic disorder of the nose triggered by allergens such as house dust mites, pet dander, or pollen. Allergic rhinitis has become a major health problem worldwide and affects a substantial number of people. The Allergic rhinitis and its impact on asthma (ARIA)2008 document estimated that over 600 million patients from all countries, all ethnic groups, and of all ages suffer from AR [1]. An epidemiological study in 11 major Chinese cities reported that the prevalence of allergic rhinitis has increased to 24.6 % in 2007 [2]. Although allergic rhinitis comprises the classic symptoms of sneezing, rhinorrhea, and nasal obstruction, it is associated with impairments of patient function in day-to-day life. Patients with allergic rhinitis may also suffer from sleep disorders, emotional problems, and impairment in activities and social functioning [3].

Pharmaceutical care is an optimal strategy for the management of allergic rhinitis. A stepwise medical treatment was proposed in the ARIA workshop report [1]. However, not all the patients’ symptoms can be controlled with pharmacological treatment. Treatment based on guidelines is not effective in all patients [4]. Around one-third of patients with moderate/severe symptoms are uncontrolled despite optimal pharmacologic treatment [1]. Complementary/alternative treatment is now being extensively used for allergic rhinitis and appears to yield satisfactory results [5]. Among these interventions, acupuncture is widely accepted as an easily available and affordable treatment choice in China and other countries, to relieve the symptoms of AR [6–10]. Catgut implantation at acupoints is a subtype of acupuncture. In this treatment, approximately 1- to 1.5-cm-long catgut is embedded in the acupoint by a special needle. The catgut can be completely absorbed by the tissue in 2–4 weeks. Then, the continuous stimulation caused by the catgut at the acupoint produces a therapeutic effect. Therefore, catgut implantation at acupoints may help treat chronic diseases such as AR [11–13].

We carried out a systematic review in order to assess the effectiveness and the possible adverse effects of catgut implantation at acupoints for allergic rhinitis [14]. In this review, due to the methodological shortcomings and risk of bias of the included trials, limited evidence showed that catgut implantation can be used to treat allergic rhinitis. There is an urgent need for robust randomized clinical trials (RCTs) with high quality and larger sample size in this field.

Therefore, we undertook a randomized, double-blind, large-scale study to evaluate the effectiveness of catgut implantation in adults with allergic rhinitis. This study has been approved by the Sichuan Regional Ethics Review Committee on Traditional Chinese Medicine (ethics approval number, 2012KL-002). The work reported in this article has been registered with an identifier (ChiCTR-TRC-12002191) by the Chinese Clinical Trial Registry.

## Methods

A detailed description of this double-blinded randomized clinical trial with two parallel groups (described as “treatment group” or “sham-controlled group” respectively) has been previously published [15]. This study protocol followed the guidelines for clinical research on acupuncture (WHO Regional Publication, Western Pacific Series No.15, 1995) and both the recommendations of Consolidated Standard of Reporting Trials [16] and Standards for Reporting Interventions in Clinical Trials of Acupuncture [17]. The protocol was approved by the Sichuan Regional Ethics Review Committee on Traditional Chinese Medicine, and the ethics approval number of 2012KL-002. The work reported in this article was registered with an identifier (ChiCTR-TRC-12002191) by Chinese Clinical Trial Registry.

### Participant inclusion criteria

The clinical diagnostic criteria published by ARIA in 2008 [1] was used to assess the eligibility for participation with AR in this study. The diagnosis of allergic rhinitis was based upon the concordance between a typical history of allergic symptoms and diagnostic tests. Typical symptoms of allergic rhinitis included rhinorrhea, sneezing, nasal obstruction, and pruritus. Prick and puncture tests were used for the diagnosis of specific allergies. Subjects were included if they met the following inclusion criteria:

1. Patients aged between 18–70 years
2. Those not participating in any other clinical trial
3. Those with no previous experience of catgut implantation at acupoints
4. Those who provide written informed consent
5. Those presenting with typical symptoms of AR, such as rhinorrhea, sneezing, nasal obstruction, and pruritus. These symptoms should last more than one hour on most days.
  Some patients may have ocular symptoms due to outdoor allergens.
6. Those with positive skin prick and puncture test performed by trained health professionals.
  Standardized vaccine (Alutard SQ, ALK- Abelló, Denmark) was used in the prick and puncture test. Oral H1-antihistamines are not permitted before the skin test. Skin prick tests should be read at the peak of their reaction by measuring the wheal and flare approximately 15 min after the performance of the test.

### Exclusion criteria

Patients with any of the following conditions were excluded:

1. Pregnant women or women who were ready to conceive in the past six months, or lactating women
2. Patients who were receiving immune therapy
3. Patients with other allergic diseases such as bronchial asthma or allergic purpura
4. Patients with nasal polyposis
5. Patients with heterologous protein allergy
6. Patients with other organic disorders such as AIDS, vascular malformation, hypertension, hematologic diseases, diabetes mellitus, malignant tumor, or mental disorders

### Recruitment

Participants were enrolled either by the recruiting hospital when they came to our otorhinolaryngology clinics to seek therapy or by the printed recruitment posters in the hospital and the campus of Chengdu University of Traditional Chinese Medicine. Patients who satisfied the inclusion criteria and were willing to participate in the trial were recruited.

### Randomization and blinding

A random allocation sequence was generated by Excel (Microsoft Office) random number generator (Microsoft, USA). Patients were assigned to either the real catgut implantation group or the sham-controlled one in 1:1 ratio by computer program. The random numbers were sealed in opaque envelopes. Participant who met the inclusion criteria was asked to pickup one of the envelopes. They were randomized into either the study or control groups depending on the random number that was in the envelope. At the same time, the corresponding randomization information of the participant was collected, which included the participant’s name in Pinyin format, the participant’s numerical birthday and gender. All this information was recorded in duplicate and sealed in opaque envelopes. These opaque envelopes were preserved separately by the principal investigator and the primary sponsor. The envelopes were all kept intact until the blinding was removed. Participants, researchers, and study physicians who interviewed and recruited patients were blinded to the group assignments. For the sake of the specialty of the treatment of acupuncture, the acupuncturists were not blinded during the study. However, they were allowed to neither communicate with the participants about the treatment nor participate in the assessment of study outcomes.

The two groups were designated A (real treatment group) and B (sham-controlled group) in the medical records and case report forms. All information was locked out and saved in a database. Blind exposure was not provided until the statistical analysis was complete. Then two opaque envelopes containing the random allocation tables were unsealed by the principal investigator and the primary sponsor simultaneously to determine which groups the participants belonged to.

### Procedures

**Fig. 1:**
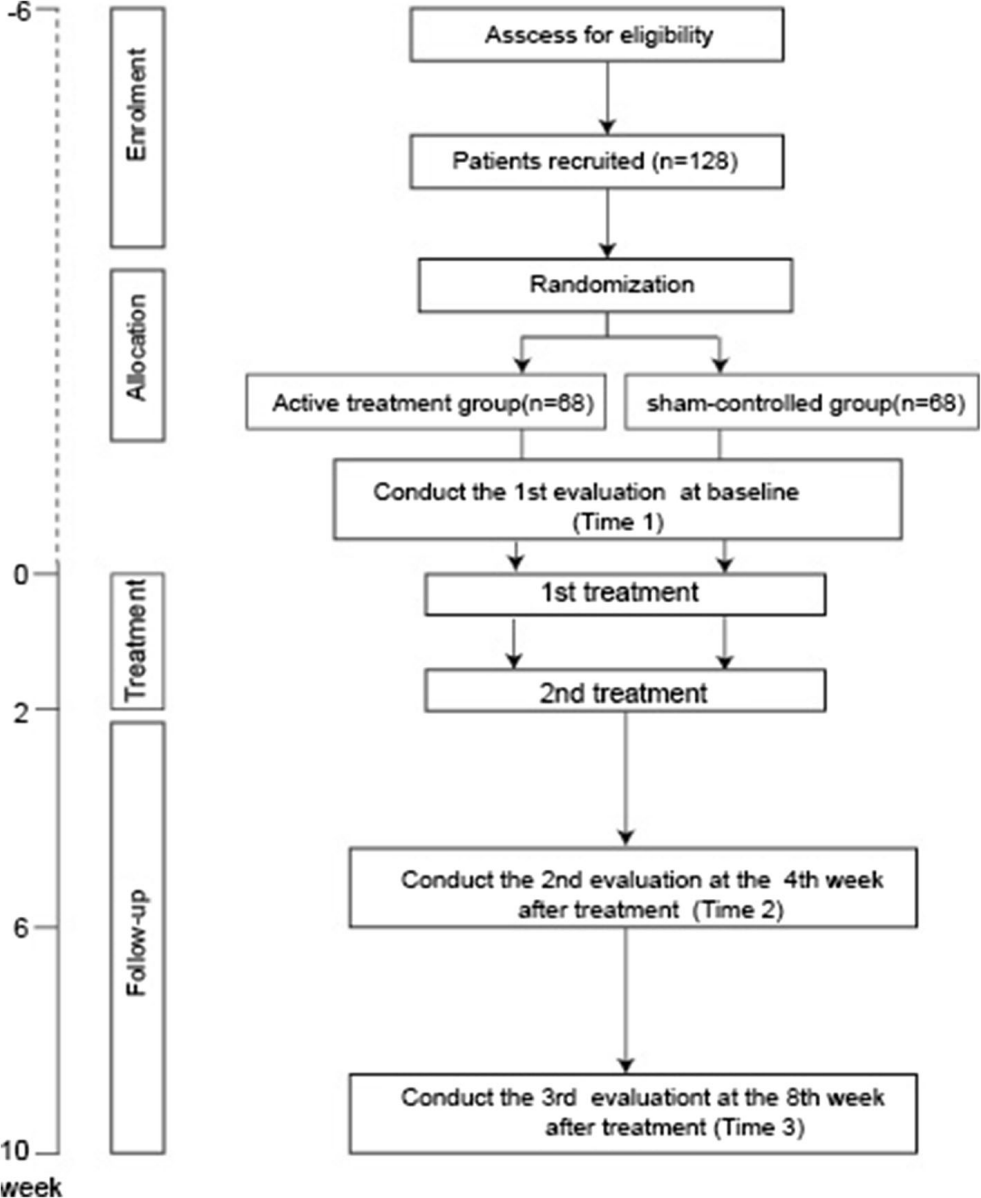
Flowchart

A total of 128 participants who were found eligible for inclusion and provided their written informed consents to participant in the study wereassigned into one of the two study arms according to the random numbers of computer-generated allocation sequences, including the sham-control group consisting of patients who would receive a placebo treatment. After completing the baseline measures, both groups received real or sham catgut implantation at acupoints once per two weeks. Each patient had 2 treatment weeks and 8 weeks of follow-up. Figure 1 illustrates the flowchart of this study. The intervention was designed according to the documentary records in the ancient books and was consistent with both the Standards for Reporting Interventions in Controlled Trials of Acupuncture (STRICTA) guidelines for the performance of acupuncture studies [18] and the World Health Organization (WHO) standard acupuncture point locations in the Western Pacific Region Geneva ( WHO,2008) [19]. Both the real and sham catgut implantation was performed by the same acupuncturist throughout the study. Acupuncturists should have undergone at least 8 years of acupuncture training and be qualified TCM doctors.

### Intervention

**Fig. 2:**
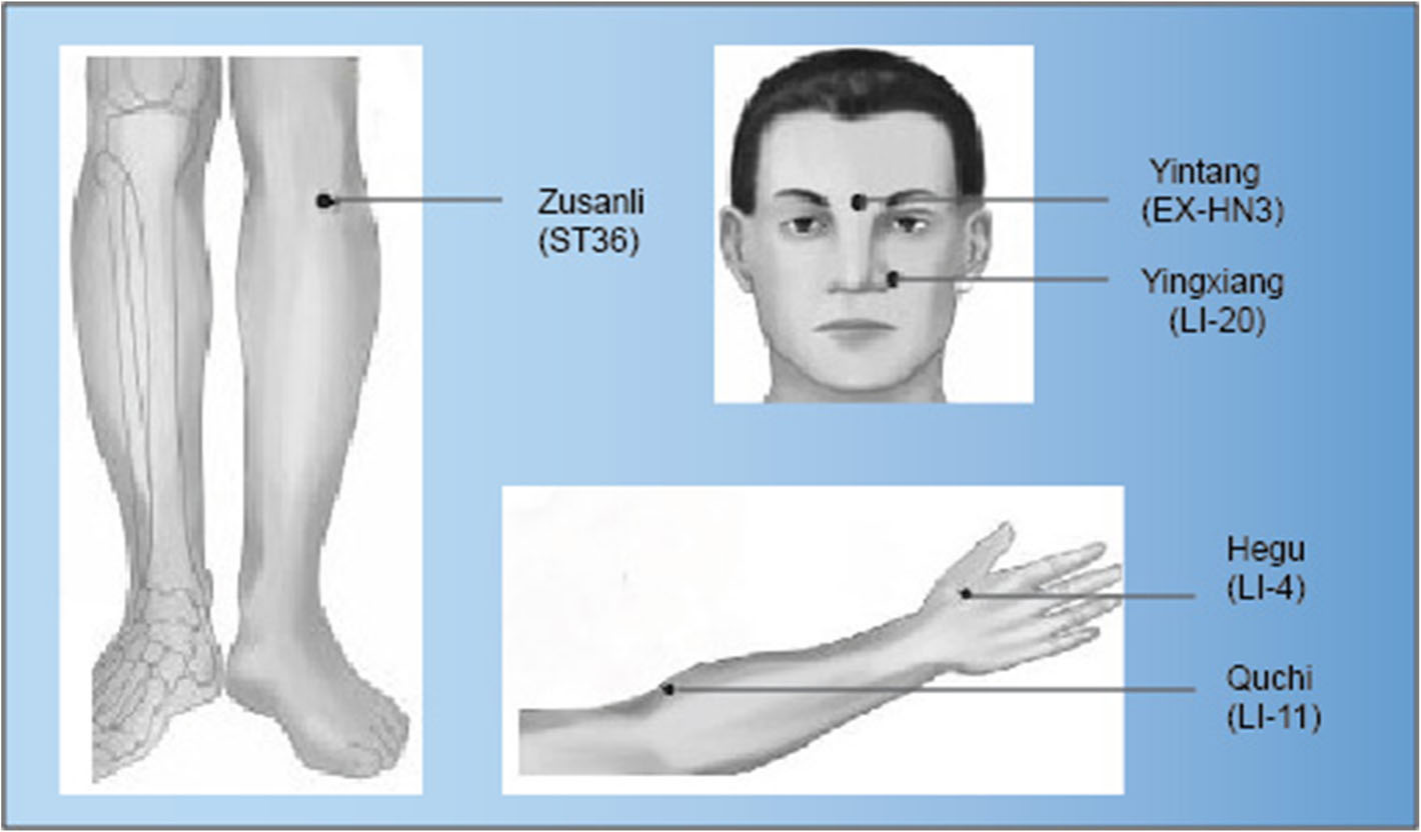
Locations of selected acupoints (for both of the groups)

Five acupoints were selected for this research on the basis of our previous study [20]. For the first treatment, three acupoints were used for each patient. They were Yingxiang (LI20), Yintang(EX-HN3), and Hegu(LI4). For the second treatment, Zusanli(ST36) and Quchi(LI11) were chosen (Fig. 2). The acupoints were the same in both the treatment groups. All the treatments were performed by the same registered acupuncturists to ensure participant blinding and consistency of treatment.

To locate the acupoint [21]:

- Hegu (LI4): abduct thumb and index figure, the point is located at the middle point between the web and the junction of the first and second metacarpal bones.
- Quchi (LI11): with the elbow flexed, the point is located at the end of the cubital crease.
- Yingxiang (LI20): in the nasolabial groove, beside the midpoint of the lateral border of the ala nasi.
- Yintang (EX-HN3): on the forehead, at the midpoint between the eyebrows.
- Zusanli (ST36): on the anteriolateral side of the lower leg, 3 *cun* inferior to Dubi (ST35), one finger breadth (middle finger) lateral to the anterior crest of the tibia. (Dubi-ST35: with the knee flexed, the point is located on the knee, in the depression internal to the patella and the patellar ligament.)

Participants received one session of real or sham catgut implantation at acupoints once every two weeks. Subjects were advised to lie supine. The pulse rate, blood pressure, and oxygen saturation were monitored routinely during the procedure as a precautionary measure. In both groups, swabs with 75 % alcohol and dry sterile cotton wool were used when withdrawing the needles. Pre-sterilized disposable needles (9#, Hanjiang Guoxiang Medical Appliance Factory, Yangzhou, China) and catguts (000, Pudong Jinhuan Medical Products Co., Ltd., Shanghai, China) were used for the treatment. For Yintang (EX-HN3), the needle was inserted to a depth of 1.0 cm downward to the nose in a horizontal direction with respect to the skin. For Yingxiang(IL20), the penetration was 2.0 cm in an oblique direction along the nasolabial sulcus towards the root of the nose with respect to the skin. For Hegu, Zusanli, and Quchi, the penetration was 2.5 cm, and carried out in a perpendicular direction with respect to the skin (Table 1). Twirling, lifting, and thrusting of the needle were performed. After treatment, the skin of the acupoints should not be touched with water or any cosmetics for three days.

**Table 1:** Acupoints and manipulation in both the real and sham groups

Weeks for treatment	Acupoints	Angle and direction	Depth
Week 1	Yintang(EX-HN3) (unilateral)	Downward to the nose in a horizontal direction with respect to the shin	1.0 cm
	Yingxiang(LI20) (both)	In an oblique direction along the nasolabial sulcus towards the root of nose with respect to the skin	2.0 cm
	Hegu(LI4) (both)	Perpendicular direction with respect to the skin	2.5 cm
Week 2	Zusanli(ST36) (both)	Perpendicular with respect to the skin	2.5 cm
	Quchi(IL11) (both)	Perpendicular direction with respect to the skin	2.5 cm

**Fig. 3:**
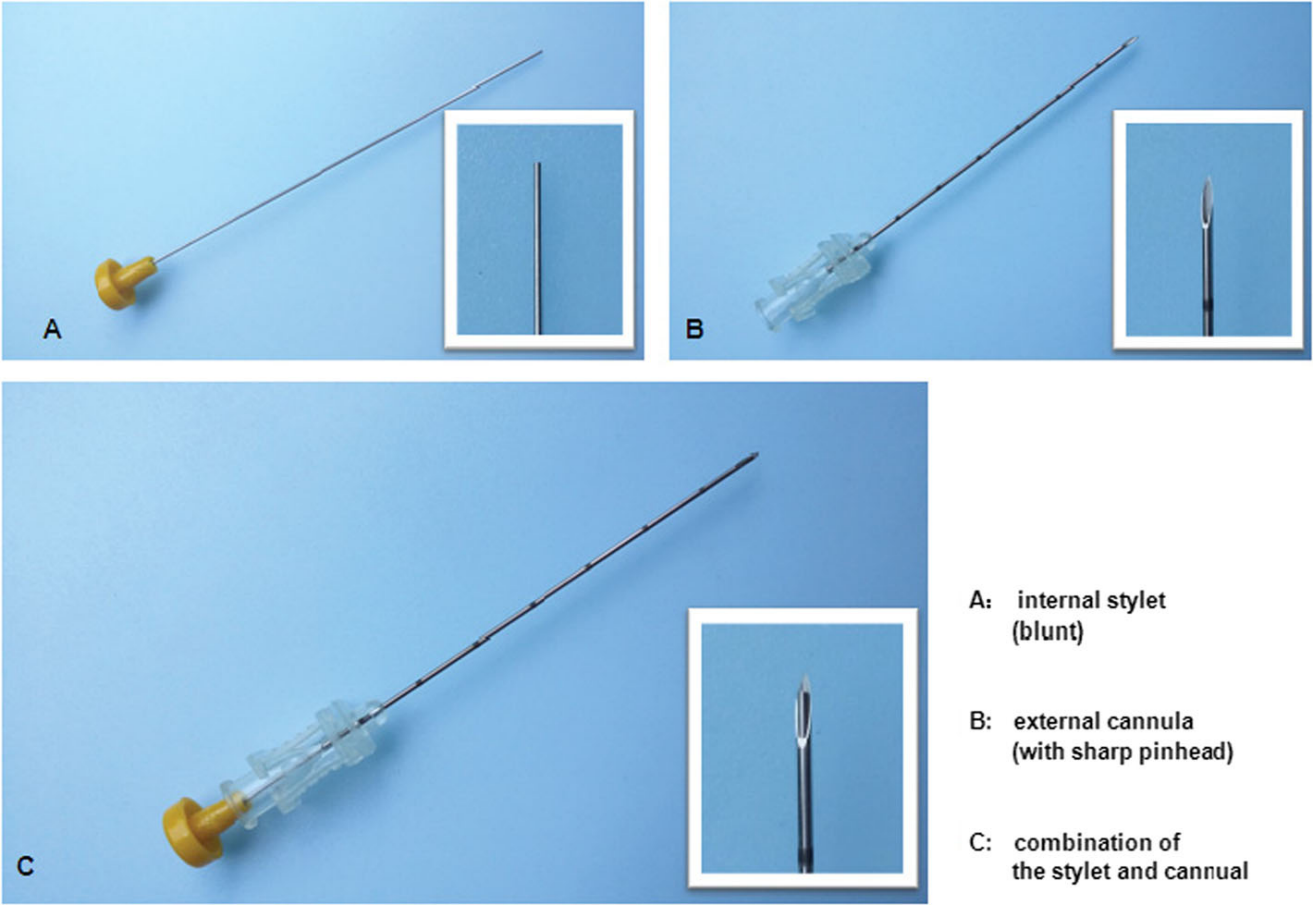
Needle used for the treatment

**Fig. 4:**
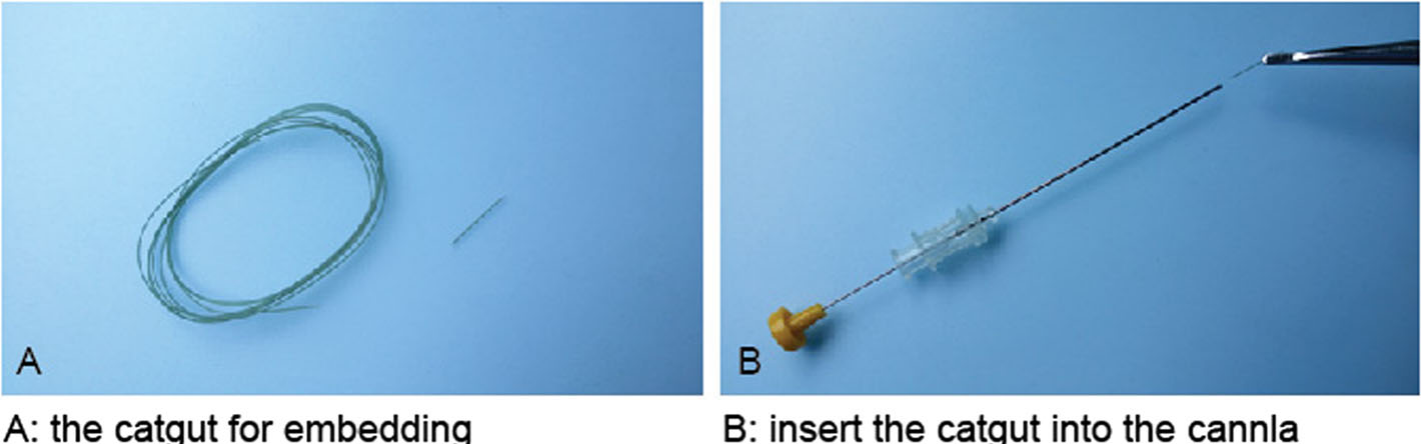
Insert the catgut into the cannula of the needle

Needle used for catgut implantation was made up of two parts: the internal blunt stylet and the external cannula with a sharp pinhead which ensured that the cannula could puncture the skin. These two parts made the needle similar to a kind of trocar. In real catgut embedding, the internal stylet should be withdrawn from the cannula for about 1.5 cm. Then a 2- to 3-mm catgut was placed into the cannula from the side of the pinhead. When the needle is inserted into the acupoints with proper depth, the catgut was pushed into the tissue of the acupoint by the stylet (Figs. 3 and 4).

For sham catgut implantation, the whole therapeutic procedure was the same as that in the real group, with the same acupoints, the same needles, and the standard techniques. However, only the sham treatment group did not contain catgut was not present in the cannula in the sham treatment. Without the catgut embedded in acupoints, patients in this group received just the stimulus of needling. To ensure the blinding, acupuncturists were instructed not to communicate with the participants throughout the duration of the treatment.

In both groups, patients were permitted to use chlorpheniramine when needed. The use of chlorpheniramine (dosage, frequency, and period of treatment) should be recorded as relief medicine scores.

## Clinical evaluation

### Primary outcome

Visual analogue scale (VAS) [22] and Rhinoconjunctivitis Quality of Life Questionnaire (RQLQ) [23, 24] were used to evaluate the severity of the symptoms of allergic rhinitis at week 0 (baseline) and at weeks 2 and 8. In VAS, the influence of the presenting symptoms of allergic rhinitis on each participant was evaluated. The symptoms were classified as mild, moderate, or severe based on the total VAS score (0–10 cm: mild, VAS 0–3; moderate, VAS 3.1–7; severe, VAS 7.1–10).

RQLQ was also used to assess the disease-associated problems of the participants in seven aspects of daily life: activities, sleep, non-nose/eye symptoms, practical problems, nasal symptoms, nose symptoms, and emotional symptoms. The score of each of the item was graded into 0–6. The total score of the seven items was calculated for every participant at baseline and at weeks 4 and 10.

### Secondary outcome

Relief medication scores and adverse events were evaluated as the secondary outcome. Participants were asked to complete diaries in the baseline and every two weeks after randomization. In the diaries, the participants recorded if H_1_-blocker (chlorpheniramine) had been taken during the treatment period, including the dosage and time of medication. In addition, adverse events (e.g., numbness, hematoma, local infection of the acupuncture sites, headache, fainting, and serious pain) were also recorded during the treatment and follow-up phases.

### Statistical analysis

The sample size was estimated from equations and our preliminary study. An improvement of no less than 25 % of the treatment group compared with the sham-controlled one was suggested. With participants recruited in the two groups in a 1:1 ratio, the following formula was used to estimate sample size [25]:

$$ n{}_1={n}_2 = {\left[\frac{{\mathrm{Z}}_{\alpha}\sqrt{2{\pi}_{\mathrm{c}}\left(1-{\pi}_{\mathrm{c}}\right)}+{\mathrm{Z}}_{\beta}\sqrt{\pi_1\left(1-{\pi}_1\right)+{\pi}_2\left(1-{\pi}_2\Big)\right)}}{\pi_1-{\pi}_2}\right]}^2 $$

In the formula, n_1_ and n_2_ represent the sample size of each group. π_1_and π_2_are the overall rates of each sample. π_c_ = (π_1_ + π_2_)/2, ***α*** = 0.05, Z_0.05_ = 1,96, ***β*** = 0.10, Z_0.10_ = 1.282. Statistical analysis was performed using 5 % significance and 90 % power, resulting in an estimated 64 patients per group.

To assess the baseline characteristics of the two groups, unpaired *t* test or *x* ^2^ test was used. In comparing the efficacy of real catgut implantation with the sham one, the chi-square test was used for analysis of categorical data. Measurement data were analyzed using the independent sample test or Kruskal-Wallis H Test. For non-normally distributed data, Wilcoxon rank-sum test was used. Statistical analysis was performed with SPSS software version 19 (SPSS Inc., Illinois) and the values were considered statistically significant at *P <*0.05. Statistical analysis was carried out in a blinded manner by qualified statisticians.

An intention-to-treat (ITT) analysis of the results was conducted based on the initial treatment assignment. Participants who strayed from the protocol (for instance, by not adhering to the prescribed intervention, or by withdrawing from the study due to adverse event or other reasons) should still be kept in the analysis. The missing data were supplemented with those obtained recently before the participant was lost to follow-up. A complete outcome data was performed for all randomized subjects.

### Patient safety

Before randomization, the participants were asked to take routine blood, urine, liver function, blood glucose, and kidney function tests to exclude any related serious illness. When any adverse effect occurred, proper medical treatment should be given as soon as possible.

### Quality control

All acupuncturists were experienced in the technique for catgut implantation, and how to deal with adverse events. The acupuncturists also received training on how to communicate with participants and how to keep them blind to the treatment throughout the trial.

In order to maintain the quality of this trial, audits were conducted regularly on compliance with standard operation procedures every week, and the reports of the audits were presented to the chief monitor. The database was locked as soon as the data was input. All the case report forms (CRF) in paper version were properly maintained by the investigator. The reason of withdrawing or loss of follow-up of any of the participants should be clarified, and the rate should be statistically analyzed.

## Results

### Participant characteristics at baseline

A total of 128 participants with AR were recruited and randomly assigned to the active catgut implantation group or the sham-controlled group in a 1:1ratio. Only two of the patients were unable to complete the 2-week treatment. In the follow-up period, another six cases dropped out. The main reasons for the withdrawals or dropouts are listed in Table 2.

**Table 2:** Reasons for withdrawal or dropout

	Group A (Active treatment group)		Group B (Sham-controlled group)	
	Patients lost to follow-up	Reason	Patients lost to follow-up	Reason
During treatment (weeks 0–2)	1	Not adhering to the prescribed intervention	1	Not adhering to the prescribed intervention
During follow-up (weeks 2–10)	2	Too busy to complete follow-up	1	Too busy to complete follow-up
	1	Admitted to the hospital for appendectomy	2	Went abroad for business or tour

No significant differences were identified between subjects in either group regarding age, gender, underlying health status, RQLQ score, VAS score and TCM syndrome type on baseline (Table 3)

**Table 3:** Subject characteristics

	Group A (Active treatment group)	Group B (Sham-controlled group)	Statistics	*P* value
Age (years)	36.112 ± 11.495	38.083 ± 12.106	T = 1.047	0.297
Gender^a^ (males/female)	29/35	36/28	*x* ^2^=1.532	0.216
Weight (kg)	60.391 ± 9.407	59.817 ± 8.697	t = 0.177	0.860
Height (cm)^b^	166.031 ± 6.575	164.752 ± 6.731	U = 1854.5	0.356
Pulse (beats/min)^b^	75.828 ± 6.870	77.297 ± 6.004	U = 1788.000	0.214
EO (10^9^/L)^b^	0.201 ± 0.132	0.195 ± 0.139	U = 1952.000	0.647
AST (U/L)	28.141 ± 7.322	29.672 ± 9.952	t = −0.991	0.324
ALT (U/L)	21.656 ± 6.393	23.125 ± 6.646	t = −1.274	0.205
BUN (mmol/L)^b^	4.572 ± 1.540	4.988 ± 1.325	U = 1804.000	0.245
Crea (μmol/L)^b^	53.367 ± 11.068	56.094 ± 9.018	U = 1726.520	0.125
RQLQ score	83.933 ± 10.076	88.801 ± 8.872	t = −1.405	0.171
VAS score^b^	7.036 ± 1.839	6.648 ± 1.379	U = 1739.500	0.136
TCM syndrome type^a^ (number of patients)			*x* ^2^=1.030	0.794
Insufficiency of the Spleen-qi^a^	25	23	–	–
Insufficiency of the Lung-qi^a^	23	26	–	–
Insufficiency of the Kidney-yang^a^	9	6	–	–
Retention of the Pathogenic Heat of the Lung^a^	7	9	–	–

*RQLQ* Rhinitis Quality of Life Questionnaire, *VAS* Visual Analogue Scale

For each variable except gender and TCM syndrome type, the values are expressed as the means ± SD

^a^Chi-square test

^b^Wilcoxon rank-sum test for the non-normally distributed data

The categories without ^a^or ^b^were analyzed with *t* test

### Primary outcome

For the analysis based on VAS scores, the difference between the treatment group and the sham-controlled group was not significant until 8 weeks after the 2-week treatment regimen (*t* = −2.424, *P* = 0.017; Table 4). However, the RQLQ scores between the two groups after 2 weeks of treatment completion significantly differed (*t* = −2.045, *P* = 0.05), and this difference could be observed until the end of the 8-week follow-up (*t* = −2.246, *P* = 0.033).

**Table 4:** Results on RQLQ and total VAS scores between the two groups (means ± SD)

		Total VAS score			RQLQ score	
	T1	T2	T3	T1	T2	T3
Treatment group (*n* = 64)	7.036 ± 1.839	5.302 ± 1.86	3.75 ± 1.93	83.933 ± 10.076	78.67 ± 8.57	73.73 ± 10.48
Sham-controlled group (*n* = 64)	6.648 ± 1.379	5.204 ± 1.72	4.535 ± 1.724	88.801 ± 8.872	84.471 ± 6.883	79.807 ± 6.221
Statistical analysis	U = 1739.500	*t* =0.324	*t* = −2.424	t = −1.405	*t* = −2.045	*t* = −2.246
*P* value	0.136	0.751	0.017	0.171	0.050	0.033

T1, baseline at subject recruitment

T2, 2 weeks after intervention

T3, 8 weeks after intervention

In the self-control study, the efficacy of the active treatment and sham groups was evaluated. The results indicated a significant improvement in the VAS and RQLQ scores of both the groups at weeks 4 and 8 after the treatment regimen when compared with the respective baseline scores. These differences are illustrated in Table 5.

**Table 5:** Efficacy of the active and sham-controlled groups in the self-control study

		Group A		GroupB	
		VAS	RQLQ	VAS	RQLQ
T2-T1	Statistical *t* value	9.887	4.314	12.599	5.951
	*P* value	*P* < 0.001	0.001	*P* < 0.001	*P* < 0.001
T3-T1	Statistical *t* value	13.153	6.287	13.659	5.892
	*P* value	*P* < 0.001	*P* < 0.001	*P* < 0.001	*P* < 0.001

T1, baseline at subject recruitment

T2, 4 weeks after intervention

T3, 8 weeks after intervention

### Secondary outcome

None of the participants took H_1_blockers (chlorpheniramine) as relief medicine.

No adverse events occurred that necessitated withdrawal of participants from the trial. The reported events included minor discomfort at the needling sites and subcutaneous induration (Table 6).

**Table 6:** Reported events associated with real and sham treatment participants

Group	Cases	Adverse events	Degree	Measurement	Prognosis of the adverse events
Group A	3	Subcutaneous induration the size of a grain of rice at Yingxiang(LI20) or Yintang(EX-HN3)	Mild	Hot-wet compression at the acupoint	The subcutaneous induration gradually disappeared 2–4weeks after the treatment
	1	Ache in the leg after catgut implantation at Zusanli(ST36)	Mild	Hot-wet compression was applied at the acupoint 24 h after the treatment	Cured
Group B	3	Ache in the leg after catgut implantation at Zusanli(ST36)	Mild to moderate	Hot-wet compression was applied at the acupoint 24 h after the treatment	Cured

## Discussion

This study evaluated the effects of catgut implantation at acupoints of allergic rhinitis in two parallel samples. The results showed that both the real and sham treatments were effective for alleviating the symptoms of allergic rhinitis and improving the quality of the life of the participant. However, the VAS and RQLQ scores did not improve until week 4 of the interventions, suggesting that the regulatory effect of the treatments for allergic rhinitis appeared slowly. Second, the VAS and RQLQ scores between the two groups significantly differed from the baseline after 4 or 8 weeks of treatment completion. Twelve weeks after the end of the treatment, the decreases from baseline in the TNSS (total score for the four symptoms) was greater in the treatment group than in the sham group. The improvement in the TNSS and RQLQ with treatment and the persistence of the effect appear to be the most clinically significant findings of the study.

To our knowledge, no other randomized controlled trial of catgut implantation has been reported in English, although many recent reports in Chinese literatures have reported the efficacy of this intervention for allergic rhinitis. Catgut implantation is a subtype of acupuncture that can extend the sensation of needling because of the persistent stimulus to acupoints caused by the embedded catgut. Furthermore, catgut implantation has an effect which is similar to pricking blood therapy. It was found to have a therapeutic effect for chronic diseases by dredging the channels, invigorating the pulse, and regulating the qi and blood. Our study demonstrates positive outcomes of both active and sham catgut implantation for allergic rhinitis. Although no catgut was embedded in the acupoints in the patients receiving sham treatment, the penetrating of the needles produces a therapeutic stimulation similar to that in conventional acupuncture. The external diameter of the needle (9#) used in the treatment is approximately 0.9 mm, which is larger than that used in acupuncture. The thicker the needle, the greater is the intensity of stimulation. Therefore, the participants in the controlled group received both the acupuncture stimulus and pricking blood therapy. This might explain the improved scores of VAS and RQLQ in the sham-controlled group. However, the improvements in the sham group were inferior at weeks 4 and 8 after treatment compared to the treatment group. This difference might be due to the slow but lasting stimulation of catgut implanted at the acupoints.

Deciding on an appropriate control procedure for clinical studies on catgut implantation at acupoints is a particular challenge. For the clinical trial of acupuncture, two approaches for sham-controlled treatment have been to use non-penetrating needles [26, 27] or needles inserted shallowly 1–2 cm away from the defined acupoints [28, 29]. Based on the special structure of the needle for catgut implantation, we found that the best approach for the sham treatment was to insert needles at the same acupoints with the same acupuncture manipulation but without embedding catgut. The difference between the two groups depended on whether or not the catgut was embedded. Consistent with the findings of our previous study on catgut implantation for allergic rhinitis, we found that the participants could be blind adequately by this approach. The sham treatment may have unspecific physiological effects of needling or just have placebo effects. In both groups, minor and minimal adverse events occurred only in a few cases, none of which were serious and did not result in participant withdrawal from the trial.

In our study, five acupoints were chosen for the treatment: Yintang (EX-HN3), Yingxiang (LI20),Zusanli (ST36), Hegu (LI4), and Quchi (LI11). The selection of acupoints was guided by Chinese traditional medicinal theory. In traditional acupuncture theories, allergic rhinitis is stated as Biqiu, which is in close correlation with the Taiyin lung meridian of hand and Yangming large intestine meridian of hand. Thus, the most frequently used specific acupoints for these two meridians would be selected in this trial according to a previous review of ancient and modern literature. The ancient Chinese also believed that Yangming is a meridian abundant of blood and Qi and is the acquired foundation. Four of the five acupoints selected in the study are on the Yangming Meridian, namely, Yingxiang (LI20),Zusanli (ST36), Hegu(LI4), and Quchi (LI11). Hegu(LI4) is the source-point of the large intestine meridian of the hand. Qi of the visceral is input at this point. Based on the theory in TCM, the lung opens at the nose and the large intestine which are interior-exteriorly related. Therefore, Yingxiang (LI20) and Hegu (LI4) are important acupoints for improving lung air (qi) deficiency syndrome. Zusanli (ST36) and Quchi(LI11) are sea points of Yangming large intestine meridian of the hand and Yangming stomach meridian of the foot. These acupoints are used to fortify the spleen and replenish the Qi. At the same time, stimulation of these points could reinforce earth to generate metal and regulate asthenia-cold in the remission stage of allergic rhinitis.

On the other hand, the nose is an orifice with lucid Yangin traditional Chinese medicine. Lucid Yang is confluent at the acupoints of Yingxiang (LI20) and Yintang(EX-HN3). These two acupoints are on the median line of the face and play an important role in clearing the nasal passages. In addition, Yingxiang (LI20) is considered a specific point on the Yangming large intestine meridian of the hand. In our previous trial, it has been suggested that symptoms of allergic rhinitis could be effectively alleviated by embedding catgut at these acupoints.

Not many studies have investigated the mechanism of catgut implantation at acupoints. We explored the probable mechanism focusing on neurogenic inflammation. Some studies have demonstrated that nerve stimulation could induce leukocyte activation and plasma extravasation, which is termed neurogenic inflammation [30, 31]. Based on this theory, we speculated that catgut implantation at acupoints might stimulate the sensory nerve and regulate the neuropeptide-mediated airway smooth muscle constriction and decrease vascular permeability of the nose. Our results showed that there was significant decrease in neuropeptides such as substance P (SP), neurokinin A (NKA), and calcitonin gene-related peptide (CGRP) in the nasal mucosa of catgut implantation group [32]. Hematoxylin-eosinstaining (HE) staining revealed that edema of the nasal mucosa was alleviated and the number of inflammatory cells decreased in the treatment group. This might be a preliminary attempt to understand the mechanism of acupuncture with catgut in the treatment of allergic rhinitis. Further studies are needed to elucidate the effective relationship stimulation for this treatment.

In our study, participants were permitted to take chlorpheniramine as relief medicine when they considered it necessary. As a first-generation oral H1-antihistamine, chlorpheniramine has been used to treat allergic diseases for a long time. In recent decades, it has not been recommended as the first-line medicine for allergic rhinitis as second-generation drugs are available and because of its sedative and anticholinergic effects. In our study, we desired to use short-acting relief medication that would not have a lasting effect and would only last until the participant could endure the symptoms of allergic rhinitis and stopped taking the medicine. Compared with other first-generation H1-antihistamines, chlorpheniramine has the weakest influence on the central nervous system and cardiovascular system. Furthermore, the peak concentration of this drug in plasma is reached in2.8 ± 0.8 h. The effect lasts 18–24 h. On the other hand, a longer duration of action-persistence of clinical effects could be found in second-generation antihistamines. In order to minimize the effect of the drug on the study participants, we selected chlorpheniramine as the drug of choice. For participant safety, they were advised not to drive or work at heights or engage in work involving attention to detail after taking the drug.

Although relief medicine was available as a rescue method, none of the participants took medication to relieve their symptoms. For most of the participants, the mean VAS score ranged from 5.2–8.9. In weeks 4 and 8 after treatment, within-group comparison revealed this score decreased significantly, which indicated that both real and sham catgut implantation were effective. Therefore, we believe this is why few participants sought to take relief medication during the treatment period.

There are many topics in clinical studies on allergic rhinitis. According to the severity of the disease, a stepwise treatment was proposed in the ARIA workshop, which include oral H1-antihistamines, antileukotrienes, intranasal glucocorticosteroids, intranasal H1-blocker, decongestant, specific immunotherapy, and so on. However due to the complexity of AR pathogenesis, multiple risk factors, pharmacologic treatment is not effective in all patients. Around one third of patients with moderate/severe symptoms are uncontrolled despite optimal pharmacologic treatment and some still have severe symptoms [1].

Catgut implantation at acupoints is one of the external therapies of TCM, which tries to stimulate certain portions of the body by mechanism stimulation. In fact, external therapy is also based on the differentiation of a symptom-complex and the principle behind this treatment is to invigorate or strengthen the automatic regulation capacity of the body through the meridians and collaterals.

Although the external therapy is totally different from conventional medicine, these two types of treatment should respect and complement each other. Zhou [33] integrated catgut implantation at acupoints with Loratadine tablet and Budesonide nasal spray to treat several allergic rhinitis. The result indicated a long-term improvement of severity of symptoms and signs of allergic rhinitis by the therapy. But there are various risks of biases of this trial, high level evidence could not be obtained.

Well-designed RCTs needed be conducted to provide a definitive answer for the combination therapy.

From the long-term perspective, delaying the occurrence and progression of AR and establishing an efficient and practical prevention and control system is the focus of the future AR research in the world. Possible future research direction of integrating TCM with western medication may provide a new space for the development of therapy against AR.

Although this is the first robust randomized, double-blinded large-scale clinical trial of catgut implantation at acupoints for allergic rhinitis, there are still some limitations. First, only two sessions of treatments were administrated. The duration of treatment using acupuncture with catgut is unknown. Thus, it is possible that shorter durations could have a similar effect, and that longer use of the treatment or repeated treatments may enhance the outcome, although further studies are required to verify this. Second, all participants in this study were followed up for only eight weeks. Our result just revealed a short-term efficacy of catgut implantation at acupoints for allergic rhinitis. Further research is still needed.

## Conclusion

Based on the efficacy of improving the VAS and RQLQ scores of allergic rhinitis and minor adverse events, we concluded that catgut implantation at acupoints may provide an effective and safe option for the symptomatic treatment of allergic rhinitis. This study might be the first to present good clinical evidence of this treatment in allergic rhinitis.

## Abbreviations

ALT: Alanine transaminase
AR: Allergic rhinitis
AST: Aspartate transaminase
EOS: Eosinophilia
IAR: Intermittent allergic rhinitis
ICD: International statistical classification of diseases
ITT: Intention-to-treat analysis
PER: Persistent allergic rhinitis
RQLQ: Rhinoconjunctivitis quality of life questionnaires
SPT: Skin prick test
TCM: Traditional Chinese medicine
VAS: Visual analogue scales.
